# Active Flexible Films for Food Packaging: A Review

**DOI:** 10.3390/polym14122442

**Published:** 2022-06-16

**Authors:** Ana G. Azevedo, Carolina Barros, Sónia Miranda, Ana Vera Machado, Olga Castro, Bruno Silva, Margarida Saraiva, Ana Sanches Silva, Lorenzo Pastrana, Olga Sousa Carneiro, Miguel A. Cerqueira

**Affiliations:** 1 International Iberian Nanotechnology Laboratory, Av. Mestre José Veiga s/n, 4715-330 Braga, Portugal; ana.azevedo@inl.int (A.G.A.); lorenzo.pastrana@inl.int (L.P.); 2 IPC—Institute for Polymers and Composites, University of Minho, Campus de Azurém, 4800-058 Guimarães, Portugal; b8453@dep.uminho.pt (C.B.); avm@dep.uminho.pt (A.V.M.); olgasc@dep.uminho.pt (O.S.C.); 3 PIEP—Centre for Innovation in Polymer Engineering, University of Minho, Campus de Azurém, Edifício 15, 4800-058 Guimarães, Portugal; sonia.miranda@piep.pt (S.M.); bruno.silva@piep.pt (B.S.); 4 Vizelpas—Flexible Films, S.A., Rua da Fundição, 8, Vilarinho, 4795-791 Santo Tirso, Portugal; olgacastro@vizelpas.pt; 5 INSA—National Institute of Health Doutor Ricardo Jorge, Rua Alexandre Herculano, 321, 4000-055 Porto, Portugal; margarida.saraiva@insa.min-saude.pt; 6 National Institute for Agricultural and Veterinary Research I.P., Portugal and CECA-Center for Study in Animal Science, ICETA, University of Porto, Vairão, 4099-002 Vila do Conde, Portugal; ana.silva@iniav.pt

**Keywords:** antimicrobial film, antioxidant film, food packaging, active packaging

## Abstract

Active food packaging is a dynamic area where the scientific community and industry have been trying to find new strategies to produce innovative packaging that is economically viable and compatible with conventional production processes. The materials used to develop active packaging can be organized into scavenging and emitting materials, and based on organic and inorganic materials. However, the incorporation of these materials in polymer-based flexible packaging is not always straightforward. The challenges to be faced are mainly related to active agents’ sensitivity to high temperatures or difficulties in dispersing them in the high viscosity polymer matrix. This review provides an overview of methodologies and processes used in the production of active packaging, particularly for the production of active flexible films at the industrial level. The direct incorporation of active agents in polymer films is presented, focusing on the processing conditions and their effect on the active agent, and final application of the packaging material. Moreover, the incorporation of active agents by coating technologies and supercritical impregnation are presented. Finally, the use of carriers to help the incorporation of active agents and several methodologies is discussed. This review aims to guide academic and industrial researchers in the development of active flexible packaging, namely in the selection of the materials, methodologies, and process conditions.

## 1. Introduction

Food packaging is used to enclose food products and presents as main functions to: contain and protect foods from the environment, namely gases, ultraviolet radiation, and chemical and microbiological contamination. Therefore, it has a crucial role in guaranteeing the quality and safety of food products, and their shelf-life. Food packaging can be in the form of a bag, bottle, can, box, wrapped pouch, or other type of containers and can also be used to communicate with the consumer and be used as a utility. The food packaging industry has suffered significant changes over the years, and new materials and technologies have been developed to reach the industry and consumers’ demands.

Currently, various approaches, such as modified atmosphere packaging (MAP) and active packaging, prevent or reduce food product damage and spoilage, and extend their shelf-life. Therefore, these approaches allow to save energy, decrease the production costs, protect the sensorial and nutritional quality of food, and protect the consumer’s health [1,2]. Moreover, intelligent or smart packaging has been presented as a unique technology to help industry and consumers to monitor the quality and safety of food products.

Active packaging is one of the most recent approaches used in the food packaging area. According to the European Commission (EC) Regulation No 450/2009, active packaging are systems projected “to extend the shelf-life or to maintain or improve the condition of packaged food. They are designed to deliberately incorporate components that would release or absorb substances into or from the packaged food or the environment surrounding the food”. Therefore, this type of packaging is developed to increase the shelf-life of foods while maintaining their nutritional quality and ensuring safety. Currently, active packaging continues to be explored and there is an increasing interest in its application in the food area. The active packaging interacts with the packaging environment (i.e., headspace) or directly with the food product. So, the active agent can be a scavenger, which absorbs the residual oxygen of headspace, moisture or water, and ethylene resultant from food maturation. Alternatively, the active agent can be released/emitted over time, in a controlled way, from packaging to headspace, or to the food, inhibiting the development of bacterial microorganisms, for example [1,3]. The active agent can be an individual substance or a combination of substances (EC Regulation No 450/2009). It can be a synthetic material, metal, inorganic material, salt, and enzyme, and is used according to the intended activity [3]. The interest in natural or organic materials, such as plant extracts, biopolymers, and essential oils (EOs) has increased in the past decade. These are used to substitute some synthetic materials, such as antioxidants and antimicrobials, as reported in several studies [4,5,6]. Another approach is the use of nanoparticles or carriers loaded with active agents. The nanomaterial concept is defined by the European Union (EU) in the Recommendation No 2011/696/EU, 2011. Currently, and according to EU Regulation No 10/2011 and its amendments, the use of some nanostructures is allowed in the manufacture of plastic materials for food contact.

Plastic films are one of the most popular products used for food packaging. The main materials used in the production of these films are synthetic polymers. This is due to their unique properties, such as ease of processing, transparency, flexibility, lightweight, and low cost. The most often used polymers to produce flexible and rigid food packaging are low-density polyethylene (PE-LD), high-density polyethylene (PE-HD), polypropylene (PP), poly(ethylene terephthalate) (PET), poly(vinyl chloride) (PVC), ethylene vinyl alcohol (EVOH), and polystyrene (PS), among others. Among all these materials, PE-HD and PE-LD are extensively used in film packaging [7,8]. These materials present low cost, low water vapor permeability values, high resistance to tear by presenting outstanding elongation at break values, good thermal stability and, at the same time, low heat seal temperature [9,10]. Currently, food packaging based on biopolymers obtained through the synthesis of bio-derived monomers (such as polylactide acid (PLA)), or produced by microorganisms (such as polyhydroxyalkanoates, PHAs), are being produced. However, these solutions are still not common, because they are expensive when compared with the synthetic alternatives [11,12]. There is also the possibility of producing flexible films using the biopolymers extracted from biomass, such as polysaccharides and proteins [13]. These materials have been extensively investigated for application in food packaging and some of them can already be processed in the existing conventional production lines, as, for example, the film extrusion process. However, these materials still present some drawbacks when compared with their synthetic counterparts [14]. In recent years, cellulose-based materials have been another option to substitute plastics in flexible films. This is a consequence of their low price, low weight, extensive availability, printability and good mechanical properties [12,15]. However, and since there are several reviews focused on these materials [16,17,18,19], they will not be included in the present one.

Processes such as extrusion, injection molding and thermoforming are used to produce polymer-based food packaging. The extrusion process is the major polymer processing technology in which the polymeric material is melted and shaped into a constant cross-section continuous product. Films, sheets, pipes, and profiles are some examples of extruded products. In addition, this process allows producing structures with a single layer (monolayer), or two or more layers (multilayer) using a co-extrusion process [10]. Another technique used to produce multilayer structures in food packaging is lamination. This technique is generally combined with polymer extrusion/co-extrusion and is used to produce multilayer flexible films using different materials (different types of polymers or different types of materials, such as polymers, paper and aluminum (Al)). The coating process is another technique used to produce multilayer films. Usually, the coating is used to provide the films’ aesthetic and physical properties derived from the coating material. The coating is usually applied in-line (e.g., in an extrusion line), or off-line, by spray, rolls, or dipping, to produce a thin layer on films’ surface. All these processing technologies have been used to study the addition of active agents to flexible films. It is worth mentioning that there are already some active packaging incorporating active agents in mono and multilayer flexible films produced by conventional industrial processes, as mentioned in active flexible packaging section.

Recently, two new technologies have been reported for the addition of the active agent: the impregnation of the active agents by super critical carbon dioxide (SC-CO_2_), and the loading of the active agents in carrier materials by encapsulation technologies, absorption and integration processes. These technologies proved to be efficient alternatives for preventing the volatilization or degradation of the active agent when subjected to high temperatures during extrusion. However, these strategies are still very difficult to use at the industrial scale due to the lack of devoted industrial equipment [4,20].

This review reports the recent studies about technologies used to produce active flexible films with monolayer and multilayers structures for food packaging purposes. Despite the availability of several review articles on active packaging, reporting the active agents and materials used [1,3,21], there are no reviews exploring and discussing the methodologies and processes used in the production of active packaging. This review aims to fill this gap and to discuss the methods and processes that the industry can use to produce active packaging, focusing on flexible films.

## 2. Active Flexible Packaging

Active packaging emerged in the last years in the food packaging area to prevent food spoilage and extend its shelf-life. Active packaging is produced with an active agent that interacts with the food. It is intended to prolong food shelf-life while preserving its organoleptic properties (appearance, aroma, consistency, texture, and flavor), i.e., to maintain food product quality and integrity, ensuring its safety. Active packaging systems can be divided into two groups: (1) active scavenging systems (or absorber systems) and (2) active releasing systems (or emitter systems). In the active scavenging systems, the active agent removes undesired substances from headspace, such as oxygen, moisture, carbon dioxide, ethylene, and odor, without going out of the packaging material. In the second group, the active agent is slowly released into the headspace to react with undesired substances from food products, such as reactive oxidizing species, or the active agent diffuses or migrates directly into the food.

**Figure 1:**
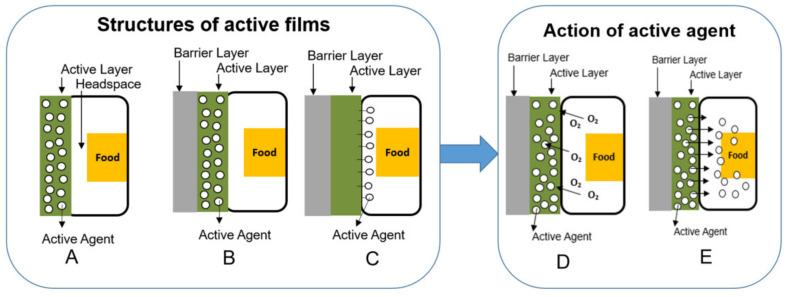
Structure of active film for active food packaging with headspace: (**A**) monolayer film with an active agent, (**B**) two-layer film with an active agent in the inner layer and (**C**) two-layer film with active substance immobilized or fixed on the surface of the film. (**D**,**E**) Schematic representation of active scavenging and releasing systems, respectively.

Active packaging has been developed wherein the active agent can be added to the packaging as an independent device (e.g., pad or sachet), incorporated into the polymeric matrix (e.g., by extrusion), or applied on the film or packaging surface (e.g., by coating). Moreover, the active agent can also be firmly fixed or immobilized on the film surface using, e.g., super critical carbon dioxide technology. Since this review is focused on technologies that allow producing flexible active films with mono- or multilayer structure, the systems where the active agent is enclosed in an independent device are omitted. Figure 1 shows a general scheme of the structures used in the production of active packaging. In Figure 1A,B the active agent is incorporated in the polymeric matrix, and then a monolayer or multilayer film is produced, respectively. The addition of layers allows decreasing the diffusion of the active agent through the film and its subsequent evaporation during storage. Sometimes, the active agent is added in the adhesive layer, used between two incompatible polymer layers, rather than in polymeric layers. In Figure 1C, the active agent is immobilized on the mono- or multilayer film surface. Then, depending on how the active agents work, they will act as a scavenger (Figure 1D) (it does not migrate) or as an emitter, migrating to the food surface or headspace (Figure 1E). The scavenging systems are used mainly to control oxygen, moisture, and ethylene inside the packaging. The releasing systems are used to confer the antioxidant and antimicrobial capacity to the active packaging. Table 1 shows the most used active agents for food packaging, their mechanisms of action and potential benefits in food applications.

### 2.1. Scavenging Systems

The oxygen scavengers (OS) are used to remove any residual oxygen present inside the food packaging or to improve its barrier properties by acting as an active barrier to this gas. They are enclosed in sachets, or incorporated into the packaging materials using, for example, the extrusion or coating processes. In the extrusion process, as presented in this review, different types of materials have been used as oxygen scavenger agents; each one has different mechanisms to react with oxygen. Many efforts have been presented in the literature, targeting to understand the oxygen scavenging processes in polymeric films and to predict their performance [22,23,24,25,26]. These processes are complex and heterogeneous, and normally involve both physical and chemical phenomena. The physical phenomenon is related to the physical dissolution and diffusion of the gas through the polymer; the chemical phenomenon is related with the reaction of the active phase with oxygen. Table 1 provides some examples of materials used and their mechanisms of action. Inorganic and metallic materials are the most used as oxygen scavengers. These compounds are stable in extreme conditions, such as high temperatures and pressures, and some are considered nontoxic. In the past decades, organic compounds extracted from natural resources, such as plants, fruits, and vegetables, have also been used as oxygen scavengers, but most of them are sensitive to extrusion temperatures. In the last years, some companies developed oxygen scavengers for incorporation in packaging materials, such as Avient (product name Amosorb™, Avon Lake, OH, USA), IPL plastics (product name ZerO_2_, Edmundston, NB, Canada), Sealed Air (product name Cryovac, Charlotte, NC, USA), and Crowne (product name Oxbar, Yardley, PA, USA).

Another type of active agent is the moisture absorber. It is used to absorb the fluids that exudate from fresh products, or to control the relative humidity inside the food packaging. The materials used for this function are commonly placed into packages in the form of sachets or pads, but there are already solutions where these agents are incorporated into the polymeric matrix used to produce trays or films. Taikous (product name Pichitto/Pichit, Gardena, CA, USA), Kyoto Printing (product name MoistCatchTM, Kyoto, Japan), and Aptar CSP Technologies (product name Active FilmTM, Auburn, AL, USA) are some examples of companies with commercial absorbing films. The most used materials in these commercial products are inorganic materials and synthetic polymers, such as silica gel and polyacrylate sodium. However, the food industry requests the development and application of natural materials or biodegradable polymers [27,29,30]. Table 1 provides examples of the natural materials most used as moisture absorbers in polymeric food packaging films. The common process used for moisture absorption in food packages is physical adsorption, but the absorption process can also happen in some cases.

The ethylene scavengers are used to remove the ethylene released during fruit ripening, thereby enhancing the fruit quality and shelf-life. Once ripening is underway, it triggers the production of more ethylene to continue the process of ripening. So, ethylene scavengers slow down the ripening process and senescence. The most used material is potassium permanganate (KMnO_4_). This is usually included in the food packaging as a sachet, but has also been incorporated into the polymeric matrix [41]. The zeolites are another good candidate to be used as ethylene absorbers, mainly due to their porous 3-dimensional structure with cation exchange, adsorption, and molecular sieving properties. These unique properties opened the possibility of using zeolites in industrial and agricultural applications, in particular as an ethylene-absorbing additive that can be used in packaging materials [33,34,35]. Other examples of ethylene scavenger materials are active clays, metals, and metallic oxides, such as titanium dioxide (TiO_2_), silver (Ag), and zinc oxide (ZnO) [32]. In Table 1, other examples are provided. There are commercial ethylene scavenging packaging materials produced by companies such as Evert Fresh (product name green bags, Katy, TX, USA) and PEAKfresh (product name PEAKfresh bag, Lake Forest, CA USA).

### 2.2. Releasing Systems

Antioxidant agents are used in food packaging to inhibit or slow down the oxidation reactions that affect food quality. They react with reactive oxidizing species (e.g., peroxides, superoxide, and hydroxyl radical) retarding or blocking the oxidation reactions of food products. The active agents are released from packaging material to headspace, by vaporization, or diffuse or migrate into the food [42,43]. Antioxidant packaging materials can be produced in the form of sachets, pads, and labels, or added directly into the films [44]. It has been shown that synthetic and natural antioxidant agents can be added to packaging systems resulting in active systems; however, for selecting an antioxidant, food characteristics and regulatory and safety issues should be considered. Synthetic antioxidants, such as butylated hydroxytoluene (BHT), butylated hydroxyanisole (BHA) and tert-butylhydroquinone (TBHQ) are already widely used by the food industry, but they are also associated with adverse side effects to human health [45,46]. Currently, the focus is on the incorporation of natural antioxidants extracted from plants and fruits, and essential oils from herbs and spices. Some examples of natural antioxidants compounds, as well as the mechanisms and potential benefits in food applications, are presented in Table 1.

Antimicrobial agents are applied to inhibit the growth of microorganisms that can cause food spoilage. Microorganisms, some of them foodborne pathogens, are the main ones responsible for food spoilage, especially in the case of fresh products (e.g., meat, fruits, and vegetables). Therefore, antimicrobial agents help to extend the shelf-life of a wide range of food products. The mechanism of action depends on the antimicrobial compounds, which can either inhibit the metabolic and reproductive processes of microorganisms, or modify the conformation of the cell wall. They are put directly in the food product, or in the food packaging as sachets and absorbent pads, or in the polymer matrix. Nowadays, antimicrobial packaging is already being used in the market. For example, the Prexelent^®^ (Rajamäki, Finland) company produces antimicrobial plastics. Recently, flexible film packaging with halloysite nanotubes (HNTs) with antimicrobial essential oils was developed in the NanoPack project, carried out in the European Union (EU). This film allows increasing the shelf-life of bread, yellow cheese, and cherries, and maintains food quality and safety standards. In the past, the most used antimicrobial agents for food packaging were synthetic materials, such as ethylene diamine tetra-acetic acid (EDTA), and metallic or metallic oxide, such as Ag, Cu, TiO_2_, and ZnO [47,48,49]. However, current developments in the industrial and scientific areas indicate that natural materials, such as chitosan, lysozyme, and citric acid, are efficient and safe for food contact [39,50,51]. More examples of this type of materials can be found in Table 1.

## 3. Methodologies for the Production of Active Flexible Packaging

### 3.1. Direct Incorporation of Active Agents in the Polymer Film Matrix

**Figure 2:**
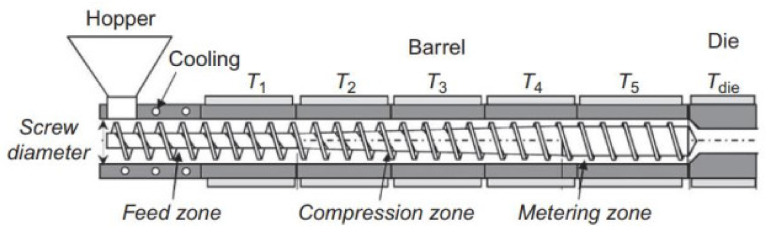
Schematic of a conventional extruder—single screw extruder. Reprinted from Covas, J., & Hilliou, L. (2018). Chapter 5—Production and Processing of Polymer-Based Nanocomposites. In M. Â. P. R. Cerqueira, J. M. Lagaron, L. M. P. Castro, & A. A. M. de O. S. Vicente (Eds.), Nanomaterials for Food Packaging (pp. 111–146) [10]. Copyright (2018), with permission from Elsevier.

Film extrusion is the most used process for the production of plastic packaging. It consists of an extrusion line that can include one extruder, or several (in the case of co-extrusion), a die and equipment to stretch/blow, cool down, cut or wind the extrudate. The process starts at the extruder throat where the material, in granular form, is fed to the screw. The rotating screw forces the polymer granules ahead in the extruder barrel, which has several controlled heating zones (Figure 2).

**Figure 3:**
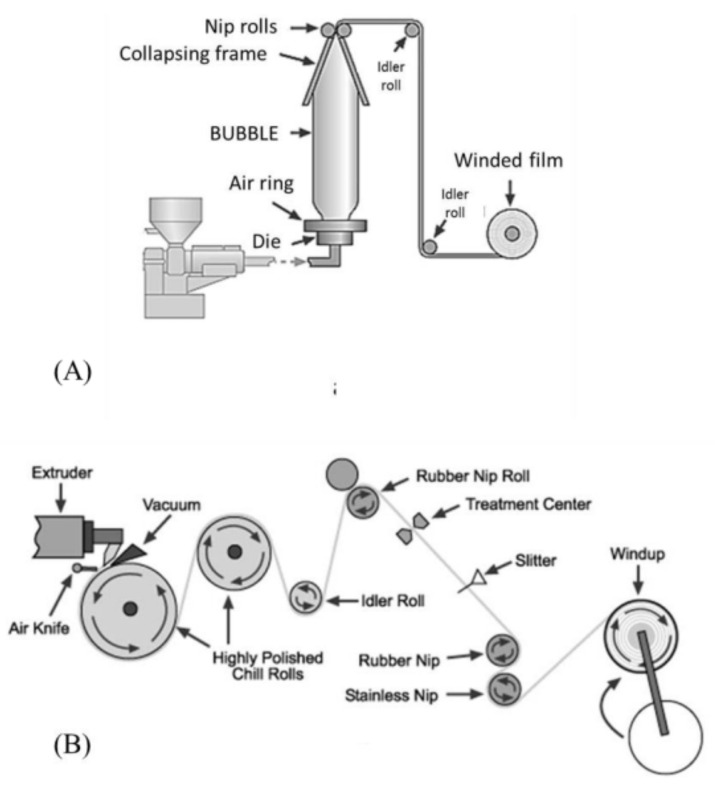
Schematic of the film extrusion/co-extrusion lines: (**A**) blown film extrusion line and (**B**) cast film extrusion line. Reprinted from Covas, J., & Hilliou, L. (2018). Chapter 5—Production and Processing of Polymer-Based Nanocomposites. In M. Â. P. R. Cerqueira, J. M. Lagaron, L. M. P. Castro, & A. A. M. de O. S. Vicente (Eds.), Nanomaterials for Food Packaging (pp. 111–146) [10]. Copyright (2018), with permission from Elsevier.

The material experiences progressive heating and pressure until melting. This allows the polymeric granules to slowly melt, reducing the risk of overheating, which can result in polymer degradation. After melting, the polymer passes through the die, which shapes the melt into an initial high thickness geometry (annulus, in the case of blown film). Afterwards, and in the blown film case, this thick annulus is stretched in the two main directions: in the machine or longitudinal direction, by the action of the pulling rolls; in the transversal or circumferential direction, by the effect of compressed air inflated through the die. The cooling of the resulting film bubble is carried out by forced air, blown through the air ring (Figure 3A). When films are directly produced in the flat shape, the extrusion die has a rectangular shape, and the film is only oriented in the machine direction; in this case, the cooling process is promoted by direct contact with the chill rolls, as presented in Figure 3B. The conventional extruders used in these extrusion lines are usually of the single screw type (Figure 2). Another type of extruders, having two screws, usually co-rotating and intermeshed (twin-screw extruders), are used for compounding purposes (e.g., to produce polymer blends, to incorporate additives/active agents, to prepare composites and masterbatches). This type of extruders are, therefore, used to prepare the compounds that are fed to single screw extruders, for the production of the final film.

The extrusion process allows the production of blown or flat films with one or more layers (mono and multilayer films, respectively). In the multilayer case, the process used is called co-extrusion, and involves more than one extruder (at least one for each different polymer system to be included in the structure of the film). The various polymers are extruded through a single die, constituting a single multilayer structure at its exit. The remaining downstream equipment is similar to that of a conventional extrusion line.

**Figure 4:**
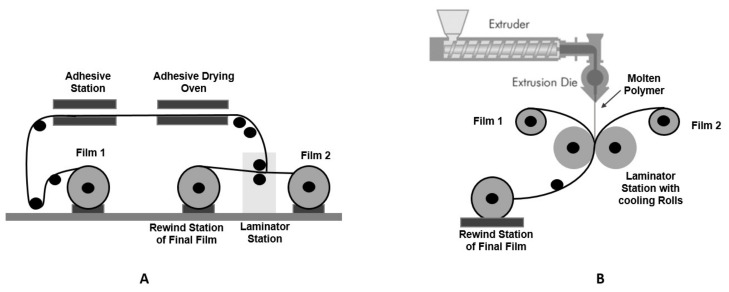
Schematic of the (**A**) lamination process outside the extrusion line and (**B**) lamination extrusion.

Another process used to produce multilayer films for flexible packaging is the lamination process, illustrated in Figure 4. This process combines individual films, which can be polymeric or non-polymeric, into a multilayer structure. Polymeric adhesives (water or solvent-based) are used to bond the different layers. This process can be used in or outside (Figure 4A) an extrusion line. Another possibility is the lamination by extrusion, wherein the extruder provides the molten film that acts as adhesive (Figure 4B). In order to improve the adhesion of the substrates, a treatment on the film surface, such as corona, can be incorporated in the extrusion line.

Multilayer films have been used for increased barrier properties. Due to the multilayer structure, they can reduce the permeation of gases through the film, and thus avoid changing the headspace composition of the package over time. However, according to the type of food, this strategy is not always the best solution to increase food shelf-life (e.g., fresh and oxygen-sensitive foods). When this strategy is not enough, the incorporation of active agents in one of the polymeric layers can be a solution. This section reports works where the direct addition of active agents to the polymer matrix was employed as a solution. Table 2 summarizes the details of some studies, such as materials used, the function of the developed packaging, amount of active agent added, and their main effects. The amount of the active substances migrated are also mentioned. Below, these works will be reported, emphasizing the conditions used in the extrusion processes and the parameters that can influence the activity of the active agent, such as the thickness of the films, dispersion of the active agent, and processing temperatures.

For example, Di Maio et al. [23] studied the effect of adding a polymeric oxygen scavenger (OS) (unsaturated hydrocarbon dienes—Amosorb DFC 4020) in a multilayer film using the same polymeric material, namely PET, to apply in fresh fruit. The films were produced by a co-extrusion process using a laboratory cast film extruder with a temperature profile of 285–280 °C. The OS was incorporated in the core layer and the pure PET was kept in the outer layers. Multilayer films with different thicknesses were produced. The authors reported that the active films developed showed a good oxygen scavenging capacity and a longer duration of activity time when the active internal layer presented a higher thickness. On the other hand, the oxygen scavenging rate was consistently lower when the external neat PET layers presented a higher thickness. The results are explained by the diffusion of oxygen through these layers, which needs more time for thicker samples before reacting with the active film. On the other hand, active monolayer films, also considered in this study, were saturated in a few days, which was explained by the fast reaction of the oxygen with the active compounds.

In 2013, Sängerlaub et al. [25] studied a multilayer film with different materials, namely PET, Al, and PE films, to apply in oxygen-sensitive food. The iron was used as an OS and was added to the PE inner layer. The PE active film was produced by extrusion (but the temperatures used were not specified). Afterwards, the lamination process was used to join the PET and aluminum (Al) films using an adhesive. The final structure obtained was PET/Adhesive/Al/Adhesive/PE-containing active agent without and with a sealing layer of PE. They also studied the effect of the OS layer and sealing layer thickness, showing that the thickness of these layers influenced the OS activity. In addition, they studied the effect of the addition of OS on sealing defects, such as small pinholes up to a diameter of 10 and 17 mm, and showed that the OS was able to compensate the sealing defects. Granda-Restrepo, Peralta, Troncoso-Rojas, & Soto-Valdez [52] studied the antioxidant properties of PE-HD/EVOH/PE-LD multilayer films produced by a blown film co-extrusion process (the temperatures used were not specified). Different active agents, such as BHA, BHT, and α-TOC, were added to the inner layer (PE-LD). TiO_2_ was added to the outer layer (PE-HD) to prevent light transmission through the films, avoiding the use of an Al layer in the film structure. They packaged whole milk powder to perform migration tests and the PE-LD layer with antioxidant agent was put in direct contact with the product. They showed that the structure of these films avoided the loss of antioxidants to the environment and favored the migration to the product. However, they also showed that during the extrusion process the concentration of BHA, BHT, and α-TOC decreased approximately 17, 41, and 23%, respectively, which was explained by the processing temperatures.

Soysal et al. [8] studied the antimicrobial activity of PE-LD/PA/PE-LD multilayer packaging film using different antimicrobial agents, such as nisin, chitosan, potassium sorbate, or silver substituted zeolite (AgZeo). In addition, the authors selected different polymers to combine different barrier properties, namely a good barrier to water vapor that comes from PE-LD and a good barrier to gases that comes from PA. The multilayer film was produced by a blown film co-extrusion process (temperatures used were not specified). The drumsticks were the product selected for the study and singly vacuum-packaged in active films developed, it means that the active agents acted by direct contact with product. The results showed that the incorporation of the antimicrobial agent was an asset to avoid the microbial growth and increase the shelf life of product. They showed that the nisin and chitosan were among all those that reduced the levels of antimicrobial activity. However, the addition of these antimicrobials increased the cost of food packaging (not more than 2%), but they mentioned that this could be compensated by the benefits of increasing the food shelf-life.

Nowadays, the use of multilayer films is common among food packaging solutions, but there are few publications about active packaging based on this type of films. This is probably related to the lack of laboratory co-extrusion lines or to the difficulty in using the high throughput industrial co-extrusion lines. On the other side, there are a lot of studies about active packaging in monolayer films; some of them are presented below and in Table 2. There are also studies where the extrusion process was only used to produce compounds, being the films produced by compression molding in a hydraulic press. Since these films are not representative of the industrial ones, these studies were not considered in the present review. As with all multilayer active films, most of these works evaluated the development of antimicrobial and antioxidant activity of films.

Beigmohammadi et al. [47] studied the antimicrobial activity of PE-LD film loaded with Ag, Cu, and ZnO. The authors selected metallic nanoparticles to study their effect on microorganisms’ growth in cheese, since these NPs have significantly reduced the microbial population in other products. PE-LD/NPs blends were produced using a twin-screw extruder, and different compounds were developed. Afterwards, the compounds were processed in a cast film extrusion line and active monolayer films were produced, using a temperature profile of 185–239 °C. Cheese samples were packaged with these films. However, the type of the packaging used (direct contact or headspace) was not specified. Of all active films developed, the one incorporating CuO was the one showing the lower coliform load of the cheese, and not showing any toxicity. Moreover using metallic nanoparticles, Li et al. [49] studied the antimicrobial capacity of PE-LD with Ag/TiO_2_ nanopowder against *Aspergillus flavus* and the mildew. First, they prepared a masterbatch incorporating Ag/TiO_2_ in a PE-LD matrix, using a twin-screw extruder. This masterbatch was later diluted in more PE-LD for the production of a flexible film, by the blown film extrusion process. The temperature profiles used in these extrusion processes were not provided. After, they studied the antimicrobial activity of the films and performed the migration test of Ag^+^ ion using rice. The type of packaging used (direct contact or headspace) was not specified. The results showed that the small amount of silver migrated from the active films inhibited the *A. flavus* significantly and reduced the mildew of rice during storage. Emamifar et al. [53] also studied the effect loading different particles in a PE-LD film, such as P105 powder (with TiO_2_ + Ag NPs) and ZnO NPs, on antimicrobial activity in packaging of fresh orange juice (packaging with direct contact with product). They produced the compounds in a twin-screw extruder, after they used a blown film extrusion line to produce a monolayer flexible film using a temperature profile of 60–175 °C. They reported that increasing the ZnO NPs concentration up to 1 wt.% caused the NPs agglomeration during the processing and this decreased the antimicrobial activity of the film. To reduce the tendency for NPs agglomeration, Emamifar & Mohammadizadeh [54] used a compatibilizer, namely polyethylene-grafted with maleic anhydride (PE-g-MA), in the preparation of the blends. They obtained a better dispersion of NPs even increasing their concentration for 3 and 5 wt.% This procedure resulted in a considerable increase in the antimicrobial activity of the film.

When the active agents are sensitive to the temperature and easily released, such as natural extracts or essential oils, some authors have tried some specific strategies. For example, Zhu, Lee, & Yam [55] incorporated α-TOC (3000 mg/kg) into the PE-LD/PP blends using a single-screw extruder, at 221 °C, and produced the PE-LD/PP blends monolayer films with antioxidant proprieties. They reported that 90% of α-TOC incorporated into the films was retained after the extrusion process. Concerning the release of α-TOC, the results showed that the higher the PP ratio in the blend the slower was the α-TOC release. The authors explained that this happened likely due to the more tightly packed structure and higher crystallinity of PP when compared to LD-PE. Graciano-Verdugo et al. [56] added 20 and 40 mg/g of α-TOC into pure PE-LD. First, they pre-mixed manually the component at room temperature, and then the blown film was produced at 165 °C, using a pilot size single-screw extruder. Even without using high temperature during mixing, it was not possible to avoid the losses of 5 and 25% α-TOC in films with 20 and 40 mg/g, respectively, after the extrusion process. However, and in both cases, the antioxidant capacity of the film was still observed in corn oil, where the active packaging acted by direct contact with the food product.

Biodegradable polymers have also been used to develop active film packaging, since many of them can already be used in conventional polymer processing technologies. For example, Llana-Ruiz-Cabello et al. [40] developed an active film with PLA containing Proallium as an active agent to produce films with antioxidant and antimicrobial properties. They made bags with the developed films and stored the iceberg salad within a modified atmosphere in some studies. Different concentrations of Proallium were incorporated into the PLA matrix and active films were obtained by extrusion using a twin-screw extruder at temperatures ranging between 200 and 205 °C. The Proallium was introduced into the extruder through a lateral barrel port where the polymer matrix was already molten to reduce its possible volatilization and degradation. They reported that Proallium alone lost around 80% of weight at temperatures up to 150 °C, but when Proallium was added to PLA, no films were formed. The results showed a great antimicrobial activity with the highest concentration of Proallium, such as 6.5 wt.%, and did not show antioxidant activity. Concerning the optical properties of the film produced, it was observed that the Proallium reduced its transparency, but no significant visual differences were observed. The authors did not mention if the films have the characteristic odor of Proallium. Manzanarez-López, Soto-Valdez, Auras, & Peralta [57] used also PLA as polymeric matrix and added 3% *w*/*w* of α-TOC to produce a film with antioxidant properties. After the production of the compounding with a twin-screw extruder, the film was produced by blown extrusion process (pilot plant size extruder) using the same temperature profile of 165–170 °C. The concentration of α-TOC decreased to 2.58 wt.% after compounding, but after the film production the authors did not observe any loss. This happened because the film was immediately cooled after blowing, while the filament of the compounding was cooled at room temperature during 10–15 min. The PLA film produced with α-TOC showed a yellowish appearance. This difference was not perceptible to the naked eye in the single film, but perceptible in the film rolls. The authors did not explain the origin of the yellow color, but it probably originated from the high concentration of α-TOC used. Cestari et al. [58] developed an active biodegradable film with the addition of oregano essential oil (OEO) and potassium sorbate into TPS and PBAT (commercial name Ecoflex^®^). These mixtures were made using a twin-screw extruder with five heating zones (with a temperature profile of 90 and 120 °C). Films were produced by blown film extrusion using a temperature profile of 115–120 °C. Then the antimicrobial and antioxidant effects were studied in frozen chicken steaks stored with the film developed, but the authors did not specify the type of packaging (direct contact or headspace). They reported that the films reduced the risk of pathogen contamination, delayed the oxidation process of chicken meat, and extended its shelf-life.

Studies on ethylene scavenger and moisture absorber systems, with direct incorporation into polymer matrix and applied in monolayer packages, are scarce in the literature. For example, Tas et al. [32] studied the ethylene scavenging capacity of halloysite nanotubes HNTs-loaded PE-LD films. The incorporation of different concentrations of HNTs into PE-LD was performed using a twin-screw extruder. The film was produced in a blown film extrusion line with a temperature profile of 165–185 °C. To study the effect of these films, some products, such as bananas and tomatoes, were selected and tested, but the type of packaging (direct contact or headspace) was not specified. The authors observed that HNTs had an effect on the slowdown of the ripening process of bananas and on the retention of the firmness of tomatoes. Another example was presented by Sängerlaub et al. [59] that developed an active film with moisture absorber properties. They blended NaCl crystals with PP polymer using a twin-screw extruder with a temperature profile of 180–250 °C. The blend was used as masterbatch where the concentration of NaCl was 60% in weight. Afterwards, the masterbatch was blended (diluted) with neat PP and monolayer films were produced using a single screw extruder with a temperature profile of 180–230 °C. The results showed that the NaCl crystals incorporated in the film were able to avoid water vapor condensation in areas of reduced temperature. Moreover, the films showed an absorption capacity of water vapor around 80%.

The incorporation of active compounds through the extrusion processes can bring several advantages, namely in the production of films at the industrial typical high extrusion rates. However, extrusion is not adequate for some active compounds, such as the ones based on natural compounds, since it uses relatively high temperatures during the process. These high temperatures may lead to the degradation of the active agents, resulting in a loss of activity and change of color. Therefore, different alternative strategies have been explored to incorporate the active compounds. These will be presented in the following sections.

**Table 2:** Active agent incorporated directly in the polymer matrix.

Active Agent (AA)	Material/Matrix	Packaging Function	Processes Used	Food Product Tested/Packaging Type	Active Agent Amount	Main Effects Compared Control Film	Amount of AA Migrated *	References
Amosorb DFC 4020	PET/PET—containing AA/PET	Oxygen scavenger	Cast film co-extrusion (Temperature profile: 285–280 °C)	Fresh apple slices	10 g/100 g polymer	The multilayer films with higher thickness in internal active layer reduced the browning of fresh apple slices packaged after 15 days storage at 8 °C. This packaging also allowed preserving the initial values of the acidity and sugar content of apples.	nd	[23]
Iron	PET/Adhesive/Al/Adhesive/PE—containing AA/PE	Oxygen scavenger	Film extrusion and lamination (temperatures not specified)	Salami in a baked bread roll	-	The food samples stored 30 days at 23 °C with active film and with sealing defects of 10 mm, showed that the presence of OS was advantageous in the permanence of color of product, when compared to the packaging without OS.	nd	[25]
α-TOC and synthetic materials (BHA and BHT)	PE-HD—containing TiO_2_/EVOH/PE-LD—containing antioxidant	Antioxidant activity	Blown film co-extrusion (temperatures not specified)	Whole milk powder/direct contact	4 g of α-TOC, 4 g of α-TOC mix with 1.5 g of BHA, 1.5 g of BHT and 1.5 g of BHA (all by 100 g polymer)	The multilayer film with α-TOC in contact with whole the milk powder showed a more gradual release of α-TOC during the 30 days storage (26.8% at 30 days). In addition, this film contributed to protect vitamin A degradation presents in whole milk powder.	α-TOC–63 ± 2 µg/g α-TOC mix with BHA—64 ± 0.6 µg/g (Product stored during 30 days at 30 °C) Regulation (EU) allows a maximum of 60 mg/kg of α-TOC	[52]
Nis., Chit., PSorbate or AgZeo	PE-LD/PA/PE-LD -containing AA	Antimicrobial activity	Blown film extrusion (temperatures not specified)	Chicken drumsticks/direct contact	2 g/100 g polymer	The results indicated that the use of active bags with nisin and chitosan reduced the levels of total aerobic mesophilic bacteria (APC) and total coliform in chicken drumsticks storage during 6 days at 5 °C.	nd	[8]
NPs Ag, CuO and ZnO	PE-LD film	Antimicrobial activity	Film extrusion (Temperature profile: 180–239 °C)	Cheese/ns	1 g metal nanoparticles/100 g polymer	All active films with metal NPs showed a decline of the number of coliform bacteria of 4.21 log cfu/g after 4 weeks of storage at 4 ± 0.5 °C. The effect of each individual NPs on decreasing coliform load had the following order: CuO > ZnO > Ag.	CuO—0.23 ± 0.005 mg/kg (it was used the simulant B at 40 °C for 10 days) EFSA1 legislation allows a maximum of 10 mg of Cu/kg of food	[47]
Ag/TiO_2_ NPs	PE-LD film	Antimicrobial activity	Blown film extrusion (temperatures not specified)	Rice/ns	9 g/100 g polymer	Reduction from 7.15 to 5.48 log CFU/g in rice stored with active packaging after one month.	Ag^+^—0.0035 mg/kg (product stored 35 days at 37 °C and relative humidity of 70%) EFSA1 legislation allows a maximum of silver migration of 0.05 mg of Ag^+^/kg of food.	[49]
P105 powder (TiO_2_ + Ag NPs) and ZnO NPs	PE-LD film	Antimicrobial activity	Film extrusion (Temperature profile: 60–160 °C)	Fresh orange juice/direct contact	1.5 and 5 g of P105 powder (TiO_2_ + Ag NPs) and 0.25 and 1 g of ZnO NPs (all by 100 g polymer)	Nanocomposite film containing nano-Ag showed higher antimicrobial activity than films with nano-ZnO when they are used to pack orange juice.	5 g of P105 (Ag)–0.15 ± 0.002 µg/L 0.25 g ZnO–0.68 ± 0.002 µg/L 1 g ZnO–0.54 ± 0.005 µg/L (product stored at 40 °C for 112 days) EFSA1 legislation allows a maximum of 10 ppm of Ag Regulation (EU) allows a maximum of 25 mg of Zn/kg of food	[53]
α-TOC	PE-LD/PP blend film	Antioxidant activity	Film extrusion (Temperature profile: 221 °C)	-	3000 mg/kg	The PE-LD/PP blend films with higher PP ratio showed a longer induction period of oxidation against linoleic acid oxidation (6 days) due to the low releasing of TOC in LDE/PP blend films, allowing an antioxidant effect for more time.	nd	[55]
α-TOC	PE-LD film	Antioxidant activity	Film extrusion (Temperature profile: 165 °C)	Corn oil/direct contact	20 and 40 mg/g	Increase of shelf life of corn oil from 12 to 16 weeks stored at 30 °C.	nd	[56]
Proallium	PLA film	Antioxidant and antimicrobial activity	Film extrusion (Temperature profile: 200–205 °C)	Salad/ns	2, 5 and 6.5 g/100 g polymer	The films developed showed no significant antioxidant activity; however, they showed effectiveness during the storage time (7 days) against all microorganisms studied, except for aerobic bacteria.	nd	[40]
α-TOC	PLA film	Antioxidant activity	Blown film extrusion (Temperature profile: 165–170 °C)	-	3 g/100 g polymer	Diffusion of α-TOC to fractioned coconut oil was slower than to ethanol with 5.1–12.9% of release. Diffusion of α-TOC to soybean oil was able to decrease the induction of the oxidation at 20 and 30 °C, but not at 40 ºC.	nd	[57]
PSorbate or/and OEO	TPS/PBAT-Ecoflex^®^ blend film	Antioxidant and antibacterial activity	Blown film extrusion (Temperature profile: 90–120 °C)	Chicken steaks frozen/ns	0.5 and 1 g/100 g polymer	Active film showed a reduction of 50% in TBARS values and a delay in microbial development when using the film with OEO and PS.	nd	[58]
HNTs	PE-LD film	Ethylene scavenger	Blown film extrusion (Temperature profile: 165–185 °C)	Bananas and tomatoes/ns	1, 3 and 5 g/100 g polymer	The results showed that the presence of 5% *w*/*w* HNTs improved the ethylene adsorption capacity of PE films by 20%. Active films slowed down the ripening process of bananas during 8 days and tomatoes only decreased their firmness 16% after 10 days of storage.	nd	[32]
NaCl crystals	PP film	Moisture absorber	Cast film extrusion (Temperature profile: 180–250 °C)	-	0.03 g or 0.06 g per 1 g of film	The PP film developed with NaCl crystals showed an absorption capacity of water vapor around 0.8 g water/g film at 97% relative humidity.	nd	[31]

**Legend: nd**—not determined. **ns**—not specified if the active agent reacts by direct contact or headspace. * Amount of AA migrated from packaging to food product tested or food simulants.

### 3.2. Incorporation of the Active Agents by Coating

A coating is a thin layer formed from a single or multiple layers spread over the surface of a substrate that gives the substrate aesthetic and physical properties derived from the coating material. The most common types of coating used in the food packaging industry are varnishes applied on the outer surface of the food packaging, used to impart a clear and glossy surface. The most conventional techniques to apply coatings are spray, gap coating, slot die coating, roll coating, and gravure coating. Most of these techniques were used/are used as conventional printing techniques, but they can be adapted for other aims, such as applying active coatings on a pre-formed film.

The conventional production process of an active coating is based on the dissolution or dispersion of an active compound in a solvent or matrix that is then applied on the surface of a substrate, and dried by evaporation or crosslinking. The crosslinking can involve curing by oxidation, temperature treatment, and ultraviolet light. Before applying the coatings, it is usually needed to evaluate the application in terms of uniformity, stability, retention of the compound selected, and application cost. In addition, when the method of application is being selected, the size and shape of the substrate surface and the substrate nature must be taken into consideration. Sometimes a treatment stage is necessary (e.g., plasma, corona and ultraviolet (UV) treatments) in order to modify the substrate’s surface in order to improve the adhesion of the coating to the surface. These techniques are commonly used in gravure printing or label printing, to increase the adhesion of inks, varnishes, and adhesives to plastic food packaging [60].

**Figure 5:**
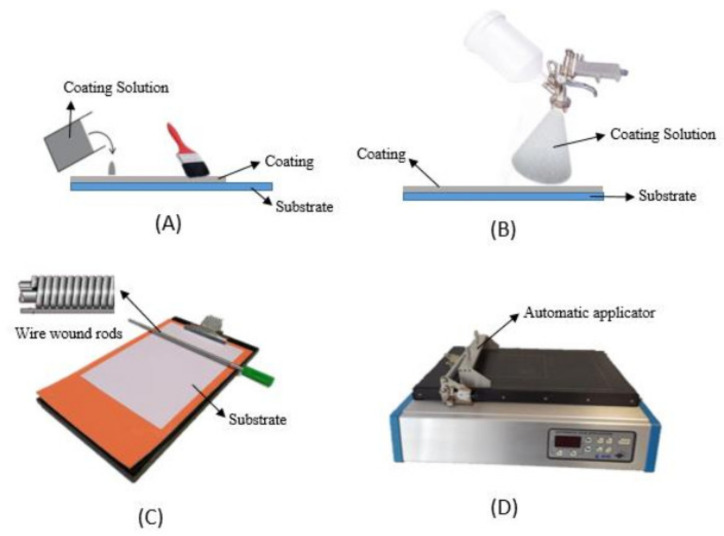
Techniques used to apply the actives coatings: (**A**) brushing, (**B**) spraying, (**C**) manual coater, and (**D**) automatic coater (or plate stripe coater).

This review reports studies on active coatings applied on the surface of flexible films, and also applied between film layers together with an adhesive. Concerning the techniques used to apply the active coatings, the most used are plate coater (manual or automatic), spraying, or simply spreading the coating over the film’s surface by brushing (Figure 5). These techniques are essentially laboratorial, but they can be easily up-scaled for industrial applications [61,62]. Table 3 presents recent works focused on the use of coatings as a strategy to incorporate active agents and their most important findings. Below, these works will be reported with an emphasis on the conditions of coatings/adhesives preparation, application processes, and main results regarding the activity of active agents.

Bolumar, LaPeña, Skibsted, & Orlien [37] tested PE-LD films coated with rosemary extract. The coating was prepared using a solution of commercial rosemary extract containing 4.5% (*w*/*v*) of carnosic acid in ethanol. They applied the coating using a brush and reached a final concentration of rosemary extract of 0.45 mg/cm^2^. The ability to counteract lipid oxidation was studied in pork patties, after 60 days of storage at 5 °C, packed in vacuum, packed (direct contact packaging) with the produced films. The rosemary extract-based active packaging showed effectiveness against the lipid oxidation when compared with the oxygen scavenging system.

Barbosa-Pereira et al. [63] studied the antioxidant activity of a coating produced with a natural extract obtained from a brewery residual waste. Different coating formulations were produced using different concentrations (3, 10, and 20% *w*/*v*) of natural extract added to a polyvinylic resin. Then the coating was applied on the PE-LD films’ surface, by plate coater, using a rod of 40 μm. The final weight was 3.2 g/m^2^. The authors studied the antioxidant activity of these coatings and compared them with a commercial rosemary extract and two synthetic antioxidants (the butylated hydroxytoluene (BHT) and propyl gallate). The effect of the natural extracts was evaluated using a headspace packaging enclosing a beef sample. The results showed that active films coated with natural extracts had an inhibitory effect on lipid oxidation and, therefore, it was concluded that they might be used to replace synthetic antioxidants.

Gaikwad, Singh, & Lee [64] studied the oxygen scavenging capacity of PE-LD films loaded with pyrogallol (PG) (a natural phenolic compound). The coating was prepared with different concentrations of PG (5, 10, and 20%, *w*/*v*) added to an ethyl acetate solution and different amounts of polyurethane. Then, the coating was applied to the PE-LD film using a plate stripe coater with a thickness of about 60—62 µm. The oxidative stability of soybean oil packed with the new films, stored during 30 days at 5, 23, and 60 °C and 95 ± 2% RH, was studied. The soybean oil showed better stabilization when packaged with PE-LD coated with 10 and 20% of PG and stored at 23 and 60 °C.

Guo, Jin, & Yang [65] developed several formulations of chitosan-based coatings (2 and 5% *w*/*v*) and tested the effect of different organic acids (acetic acid, citric acid, lactic acid, and levulinic acid, or their mixtures) in combination with antimicrobial agents, such as lauric arginate ester (LAE), sodium lactate (NaL), and sorbic acid (SA). Then, the coatings were applied on a PLA film using a brush or spray, obtaining different weights, such as 0.39 and 1.94 mg/cm^2^ of chitosan, 1.94 and 3.89 µg/cm^2^ of LAE, 0.78, 1.56, 3.8, and 7.78 mg/cm^2^ of NaL, and 0.12 and 0.23 mg/cm^2^ of SA. The antimicrobial efficacy in a microbial culture and ready-to-eat meat vacuum-packaged (direct contact packaging) was studied. In general, the results showed that the PLA films coated with chitosan containing multiple organic acids and other antimicrobials had an antimicrobial effect against *Listeria innocua*, *L. monocytogenes*, and *Salmonella Typhimurium*, showing a significant inhibition of microbial growth during 48 h at 22 °C. However, the active films developed showed to be more effective against the microorganisms in microbial culture than in RTE meat.

As mentioned above, the surface treatments of films can be used to improve the bonding of the coating to the substrate surface. For example, Al-Naamani, Dutta, & Dobretsov [66] developed a PE-LD film coated with 2% (*w*/*v*) of chitosan solution and 0.1 and 2% (*w*/*v*) of ZnO/chitosan solution. Before coating, they used plasma treatment to provide a hydrophilic PE-LD film surface. Then, they applied the chitosan solution and the chitosan/ZnO nanocomposite solution, by spray, on the PE-LD surface, and dried it at room temperature. They evaluated the shelf-life of packed okra samples during 12 days at room temperature (25 °C), but did not specify the type of packaging (contact direct or headspace). They observed that the coating developed with chitosan/ZnO reduced the fungal and bacterial growth more than the coating prepared only with chitosan. Moreover, a significant reduction in bacterial growth was observed in the samples stored in treated PE-LD film compared to the control one. Joerger, Sabesan, Visioli, Urian, & Joerger [67] applied a 2% (*w*/*v*) chitosan coating, and 5 and 4% (*w*/*v*) Ag/chitosan coating. The film, based on ethylene copolymer (EVA), was corona treated to create a reactive surface. Then the coating was spread onto the film surface using a plate coater with 28 wire wound rod. The antimicrobial activity, without and with the incorporation of Ag particles, using beef and chicken meat exudates (type of packaging not specified) was analyzed. They reported that the activity of the chitosan coating was more effective when silver was incorporated. However, the authors did not mention the effect of corona treatment.

Artibal company (Sabiñánigo, Spain) developed an active coating, described in the European Patent EP 1 477 519 A1 [68], that consists of adding the active agent to a varnish that is then applied to the films by rollers, tampography, serigraphy, or spraying systems. Using this technology, Lorenzo, Batlle, & Gómez [61] compared the effect of two natural antioxidants, such as OEO and green tea extract. These were applied at 1.5 and 2.0 g/m^2^ to a PET/PE/EVOH/PE multilayer film used to package foal steaks. Camo, Lorés, Djenane, Beltrán, & Roncalés [62] also used an oregano coating, produced by Artibal company, but applied on a PP film. The aim was to evaluate the effect of the oregano extract concentrations (0.5, 1, 2, and 4%, *w*/*v*) on the quality characteristics of stored fresh beef. In both studies, the type of packaging used was not specified. In the first study they reported that the OEO was more effective to increase the shelf-life of fresh products than the green tea extract. In the second case, they demonstrated that it is possible to increase the shelf-life of fresh products from 14 to 23 days using 2% *w/v* concentration of oregano extract.

Lee, Park, Yoon, Na, & Han [69] developed a multilayer active film, with a five layers structure, containing PP, active coating, PET, active coating, and PE-LD. The star anise essential oil (SAEO) and thymol oil (TH) were used as an insect repellent and antimicrobial agent, respectively. Different coating formulations with 25% (*v*/*v*) of SAEO and TH were prepared using ethyl alcohol, polyurethane dispersed in distilled water, and a silicone surfactant. Those active coatings were applied on different sides of the PET film using an automatic control coater, then PP and PE-LD were laminated on the SAEO and TH coating layers, respectively. The insect repellent and antimicrobial activities were evaluated using slices of bread as a food model, but the authors did not specify the type of packaging (direct contact or headspace). The film developed demonstrated efficiency against the insects and impeded the growth of microorganisms in packed sliced bread.

In 2016, Carrizo, Taborda, Nerín, & Bosetti [42] studied the performance of the flexible films (commercial bags) produced with two layers of oriented polypropylene (OPP). A special adhesive incorporating green tea extract (GTE) (system under patent protection) was used to build the multilayer. This antioxidant multilayer packaging was produced at the industrial level using a lamination process. Then, the antioxidant activity of the films and their capacity for extending the shelf-life was tested for 16 months with real food, with headspace packaging, under atmospheric pressure, in the presence of oxygen. It was demonstrated that the packaging developed protected the food against the oxidation process, significantly reducing the rancidity and, in this way, extending the shelf-life of packaged food. The migration results showed that any of the compounds of GTE, such as catechins, diffused through the polymer. This means that the free radicals released in the oxidation process diffused through the polymeric layer and arrived at the catechins location, where they were trapped and consumed. The sensorial analysis demonstrated that the packaged food was not affected by GTE. Oudjedi, Manso, Nerin, Hassissen, & Zaidi [43] also studied the antioxidant activity of two active agents (from extracts of bay and sage leaves) added to an adhesive applied between the PE-LD/PET multilayer films. First, the extracts were dissolved into isopropanol (concentration not specified) and then incorporated at 10% (*w*/*w*) into the adhesive. After, the adhesive was spread on the PET sheet using a coating machine (KK coater, RK print), resulting in a final concentration of the extracts ranging between 0.025 and 0.03 g/m^2^. Then the PE-LD film was laminated to the PET coated film using a lamination process. The authors evaluated the capacity of the active packaging with headspace to prevent lipid oxidation of fried potatoes, incubated at 40 °C for 20 days. They demonstrated that active components of extracts of bay and sage leaves also acted without migration from the material packaging to headspace, improving the shelf-life of fried potatoes. They also observed that the color of the packaging was not affected by the incorporation of the extracts.

Azlin-Hasim, Cruz-Romero, Cummins, Kerry, & Morris [70] used an innovative process to apply the active coatings. They treated PE-LD films with UV/ozone process and coated them using a layer-by-layer (LbL) technique. During the process, poly(ethyleneimine) (PEI) and poly(acrylic acid) (PAA) solutions loaded with Ag NPs were alternately deposited, producing an antimicrobial film. Another innovative solution already studied is atomic layer deposition (ALD). For example, Vähä-Nissi et al. [71] developed an active film with antimicrobial properties using ZnO and aluminum oxide (Al_2_O_3_) that were deposited at low temperature with ALD on biaxially oriented polymer films, namely BO-PLA and BO-PP.

**Table 3:** Active agents added directly to coatings.

Active Agent	Packaging Material	Packaging Function	Processes Used	Food Product Tested/Packaging Type	Active Agent Amount	Main Effects Compared Control Film	References
Rosemary extract	PE-LD films	Antioxidant activity	Brushing	Pork patties/direct contact	0.45 mg/cm^2^	The results demonstrated that PE-LD film coated with rosemary was the most effective active packaging to protect pork patties storage during 60 days at 5 °C.	[37]
Natural extract obtained from a brewery residual	PE-LD films	Antioxidant activity	Plate coater	Beef/headspace	3.2 g/m^2^	The results showed that active antioxidant films coated with natural extracts decreased lipid oxidation by up to 90% during 17 days stored at 4 °C.	[63]
Pyrogallol (PG) (a natural phenolic compound)	PE-LD films modified with sodium carbonate	Oxygen scavengers	Plate stripe coater	Soybean oil/headspace	Thickness 60—62 µm	The soybean oil samples packed with PE-LD/PG films coated with 10 and 20% PG and storage at 23 °C and 60 °C showed a better stabilizing effect during 30 days than oil packaged with pure PE-LD.	[64]
Chitosan, lauric arginate ester (LAE), sodium lactate (NaL), and sorbic acid (SA)	PLA films	Antimicrobial activity	Brushing or spraying	Ready-to-eat meat (RTE)/direct contact	0.39 and 1.92 mg/cm^2^ of Chitosan; 1.94 and 3.89 µg/cm^2^ of LAE; 0.78, 1.56, 3.89 and 7.78 mg/cm^2^ of NaL and 0.12 and 0.23 mg/cm^2^ of SA	The results showed that PLA films containing LAE were those that most significantly inhibited the growth of the tested microorganisms. The PLA films coated with NaL, and SA showed to reduce significantly the growth of L. innocua but were less effective against Salmonella.	[65]
Chitosan and ZnO/chitosan	PE-LD films	Antimicrobial activity	Spraying (and plasma treatment)	Okra/ns	Not specified	The results showed that total bacterial concentrations in films coated with chitosan/ZnO coatings were reduced by 63%.	[72]
Chitosan and Ag/chitosan	Ethylene copolymer (EVA) film	Antimicrobial activity	Plate coater (and corona treatment)	Beef and chicken meat exudates/ns	Not specified	The film coated with chitosan reduced colony counts of *E. coli* 25922 and of *L. monocytogenes* Scott A by 5 and 2–3 log10, respectively, after 24 h exposure. However, this activity was increased when silver ions were incorporated into the films that, for example, originated the complete killing of *E. coli* O157:H7 DD3795.	[67]
Oregano essential oil and green tea extract	Multilayer film: PET/PE/EVOH/PE	Antioxidant activity	Rollers, tampograph, serigraphy or spraying systems	Foal steaks/ns	1.5and 2.0 g/m^2^	The active films with essential oregano oil were significantly more efficient than those with green tea extract in case of extended fresh odor and color from 7 to 14 days, compared to the control.	[10]
Oregano extract	PP film	Antioxidant activity	Rollers, tampograph, serigraphy or spraying systems	Fresh beef//ns	Not specified	The results showed to be efficient in extending the fresh odor and color from 14 to 23 days. However, the addition of oregano should be around 1% due to the unacceptable oregano odor when the concentration is higher.	[62]
Star anise essential oil (SAEO) and thymol (TH)	Multilayer film PP/SAEO/PET/TH/PE-LD	Insect repellent and antimicrobial activity	Automatic control coater	Slices bread/ns	Thickness of active coating was 13.20 ± 1.72 µm	The developed film showed a strong and sustained insect repellent activity, lower microbial counts and better visual appearance of bread after 14 days of storage.	[69]
Green tea extract	Multilayer film: OPP/OPP	Antioxidant activity	Lamination	Dark chocolate peanuts and milk chocolatecereals/headspace	Not specified	The results demonstrated that it is possible to increase the shelf life of these products from 9 to 18 months without active agent migration from packaging.	[42]
Sage leaf (SL) and Bay leaf (BL) extracts	Multilayer film: PET/PE-LD	Antioxidant activity	Coating machine (KK coater, RK print).	Fried potatoes/headspace	0.025 and 0.03 g/m^2^	The results showed a strong antioxidant activity of SL and BL, either evaluated alone or as food packaging for fried potatoes. For example, in case of the malondialdehyde (MDA) the SL extract was more efficient, showing a reduction of 40% of MDA compared to the control, while BL showed a reduction of 31%.	[43]

**Legend: nd**—not determined. **ns**—not specified if the active agent reacts by direct contact or headspace.

### 3.3. Incorporation of Active Agents by Supercritical Impregnation

The supercritical impregnation process is a recent technique used to impregnate active agents in packaging materials. The supercritical fluids (SCFs) have been used as solvent, anti-solvent or plasticizer, in different applications, such as extraction, dyeing, cleaning, fractionation, polymerization, polymer processing, encapsulation, among others [73,74]. Supercritical solvent impregnation has been recently used to develop active packaging, as an alternative to conventional techniques. This process allows applying active agents at near ambient temperatures avoiding the degradation of the substances that are easily destroyed or deactivated by heat. Moreover, the supercritical fluid can work as a carrier of the active agent and change the matrix properties (e.g., swell), thus ensuring the impregnation success. Carbon dioxide (CO_2_) is one of the most used SCFs, since it offers several advantages, such as low toxicity, low cost, non-flammability, environmental sustainability, and it is chemically inert under many conditions [74,75].

**Figure 6:**
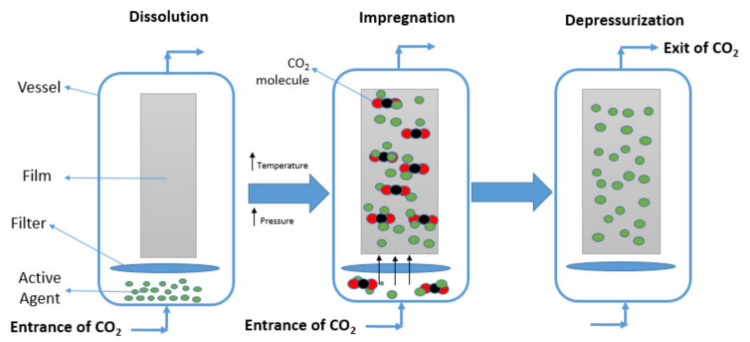
Schematic of the use of supercritical fluid carbon dioxide (CO_2_) in the impregnation process during active film production.

The impregnation process using CO_2_ fluid involves three main steps (Figure 6). Briefly, first the active agent is dissolved in supercritical fluid CO_2_ (dissolution step). Then, the dissolved active agent is transported to the surface of the material and subsequently penetrates and diffuses into the swollen polymer matrix (impregnation step). Finally, the CO_2_ molecules are removed by the shrinking polymer (depressurization step), and the impregnated molecules are trapped inside the polymer matrix. The impregnation process depends on the pressure, temperature, depressurization rate and time, variables that should be optimized [74,75].

The impregnation process can be carried out in the static, dynamic, and semi-dynamic modes. In the static mode, the active agent and film are placed in a reactor and are physically separated (e.g., metal or paper filter) avoiding the direct contact between them. Then, the dissolution of the active agent and the sorption of the mixture (active agent and SC-CO_2_) are carried out simultaneously. For the dynamic process, the dissolution of the active agent is done in the same impregnation reactor or in a previous dissolution reactor. In this case the sorption of the active agent in the film starts when the saturated mixture enters the impregnation cell. In the semi-dynamic mode, the static and dynamic modes work by time intervals. The active agent is dissolved in the CO_2_-phase in the static period, and in the following dynamic mode period the dissolved agent diffuses into the polymer matrix [75].

In the past few years, some works have shown the effectiveness of the supercritical impregnation of natural active substances, such as extracts and essential oils, in food packaging. In most of the cases, the authors studied the effect of different operation conditions, namely pressure, contact time, temperature and depressurization rate, on impregnation yield and distribution of the active substance in flexible films. In addition, they studied possible changes in the polymer thermal and mechanical properties that could result from the process, such as the high pressure and the presence of active agents. In this review, we will report the effects of the operation conditions on antimicrobial and antioxidant agents. Table 4 summarizes some of the works performed using this methodology, the materials used, and the main effects on the activity of the films.

Below, these works will be reported, mainly emphasizing the production conditions. For example, Goñi, Gañán, Strumia, & Martini [76] produced an antioxidant active film, impregnating eugenol into a PE-LLD film, and studied the effects of different operation conditions, namely pressure and depressurization rate, on impregnation yield; also the antioxidant activity of eugenol was evaluated. They reported that the best penetration of eugenol in the film was observed when high pressure and slow depressurization (pressure released) were used. However, the results of antioxidant activity of impregnated films showed a strong activity, regardless of the eugenol loading. In the case of Cejudo Bastante, Casas Cardoso, Fernández-Ponce, Mantell Serrano, & Martínez de la Ossa [77] a multilayer structure with PET/PP with antioxidant and antimicrobial agent, namely olive leaf extract (OLE) was developed. Several parameters (pressure, temperature, depressurization rate, time and presence of a modifier (ethanol) to increase the solubility of the compound in CO_2_) were studied. Then, the optimized parameters were used to impregnate the active agent OLE. Then, they applied these active films to cherry tomatoes (not specified the type of packaging applied) and evaluated them for 50 days of storage. They reported that the shelf-life of cherry tomatoes increased in the case of the impregnated films. The antioxidant and antimicrobial properties of the film were slightly higher when the OLE/polymer mass ratio of 1 and percentage of solvent of 7% ethanol was used.

Belizón, Fernández-Ponce, Casas, Mantell, & Martínez De La Ossa-Fernández [78] studied the effect of incorporation of polyphenols extracted from mango leaf in a similar multilayer film of PET/PP, and then studied the effect of their antioxidant activity in perishable food. They used the methyl gallate to determine the best conditions, namely the pressure, temperature, impregnation time, and stirring mode of the impregnation process; afterwards they used the optimized conditions for applying the mango leaf extract. Then, lettuce and tangerine were stored with the films produced (not specified the type of packaging applied) and their shelf-life was evaluated. The results showed that the films developed increased the shelf-life of the tested foods, showing that prevention of microbial contamination and organoleptic deterioration is possible.

Franco et al. [20] studied the effect of impregnation of α-TOC in single layer PP and PET, and in PP/PET multilayer films. To optimize the impregnation of α-TOC, a corona discharge treatment on PET film surface was performed. Results showed that PP films were the best option to produce a controlled-release packaging, with high values of loaded α-TOC, when compared with PET surface with corona discharge treatment. They reported that this happened due to the low penetration of TOC into the treated PET surface due to a low affinity with the additional polar groups formed during the treatment.

There are some authors that studied the effect of nanocomposite films on the impregnation process and release of an active substance. For example, Rojas et al. [79] studied PE-LD nanocomposites films with 2.5 and 5% (*w*/*w*) concentrations of an organo-modified montmorillonite (OM-MMT C20A) impregnated with thymol, using supercritical impregnation process. The aim was to study the effect of OM-MMT C20A content on the impregnation and the release of thymol, targeting its application in antimicrobial packaging. This work showed that the presence of OM-MMT C20A improved thymol incorporation in the PE-LD film, from 0.45 to 1.19% (*w*/*w*), depending on the impregnation conditions, and decreased its diffusion coefficient during the release tests, allowing its sustained release over time.

Some authors also studied this impregnation process using biodegradable polymers. For example, Villegas et al. [80] impregnated cinnamaldehyde (Ci) into PLA biodegradable films. They reported that Ci was successfully incorporated into PLA using SC-CO_2_, at higher pressure and slower depressurization rate. Results showed that PLA films impregnated with Ci had better thermal, structural, and mechanical properties than neat PLA films. In addition, the films showed strong antibacterial activity against the tested microorganisms.

From the existing examples, it can be said that supercritical impregnation is promising from an industrial point of view, and a good strategy to produce active film for food applications.

**Table 4:** Active agent sorption on the surface of the flexible films using the SC-CO_2_ impregnation process.

Active Agent	Material/Matrix	Packaging Function	Tested Product/Packaging Type	Active Agent Amount	Main effects Compared Control Film	References
Eugenol	PE-LLD film	Antioxidant activity	-	0.5 and 6 wt.%	The results of antioxidant activity showed approximately 80% inhibition after 96 h, regardless the eugenol loading.	[76]
Olive leaf extract (OLE)	PET/PP multilayer film	Antioxidant and antimicrobial activity	Cherry tomatoes/ns	2–5.5 mg/g film	The results showed that the tomatoes packed with the impregnated film did not show any physical change for the first 30 days and their appearance remained the same as at the initial moment of the experiment.	[77]
Polyphenols extracted of mango leaf extract	PET/PP multilayer film	Antioxidant activity	Lettuce and tangerine/ns	36–40 mg of total polyphenols (TP)/100 g film	The results showed that the films increase the shelf-life of lettuces for 14 days and tangerines until 39 days by preventing microbial infections and organoleptic deterioration.	[78]
α-TOC	PET film, PP film and PET/PP multilayer film	Antioxidant activity	-	2.66—3.20 mg/cm^2^ film	The results showed that it is possible to produce a multilayer film with controlled releasing of α-TOC until 14 h.	[20]
Thymol	PE-LD film/Cloisite 20A nanocomposite film	Antimicrobial activity	-	0.3—1 wt.%	They reported that the presence of nanoclays makes the release of thymol from PE-LD film difficult allowing a sustained release over time of the active compound.	[81]
Cinnamaldehyde essential oil (Ci)	PLA film	Antibacterial activity	-	72–162 mg/g film	The films impregnated with 13% of Ci showed strong antibacterial activity against *E. coli* and *S. aureus*, where no microorganism was detected.	[80]

**Legend**: **ns**—not specified if the active agent reacts by direct contact or headspace.

## 4. Processes to Incorporate the Active Agents into Carriers

In general, carriers of active compounds are materials used to transport and protect the active compounds. In the past few years, carriers loaded with active compounds have been considered an attractive method in different areas such as the pharmaceutic, cosmetic, and food industries. However, despite the wide applicability and advantages, the production processes have found little space in the food industry, mainly in food packaging area, due to its high production cost. While the pharmaceutical and cosmetic sectors often support the use of high-cost methodologies, the food industry works with lower profit margins, and therefore, production costs should be maintained low [74]. As a consequence, the use of carriers loaded with active compounds and their incorporation in food packaging has not been widely studied. Table 5 summarizes some of the works found in the literature, namely the main characteristics of the systems used, materials, amount of active agent added, and main effects on the packaging developed. Below, these works will be reported, emphasizing the production process of carriers loaded with active agents and the main outcomes.

Regarding the processes to incorporate the active agents into carriers, most of the work has been focused on the absorption in porous media materials and integration through ultrathin fibers. More rarely, the encapsulation of active compounds in hollow bodies of polymeric materials has also been studied.

In the case of the absorption in porous media materials, essential minerals, such as aluminosilicates and mesoporous silica, have been used. For example, Wrona et al. [82] used crystalline microporous aluminosilicates and incorporated green tea extract using an absorption, adsorption, or ion exchange process, as mentioned in the EP1564242B1 patent. The microporous existing on the shell facilitate the incorporation and protection of the active agents. The active materials were then incorporated into polyethylene (PE) films (not specified the type of PE), and the antioxidant activity in minced pork meat vacuum packaged (direct contact packaging) was studied. The active PE film was developed by NUREL company (Zaragoza, Spain) using an extrusion process. The results demonstrated that inorganic carriers protected the green tea extract from the adverse effects of the high temperature used in the extrusion process. Moreover, it was reported that the referred active packaging extended the shelf-life of meat and preserved its red color. The results showed that the evaluated shelf-life parameters of packed foods were better in samples packaged with active films than the ones packed with the control film. In the case of Gargiulo et al. [83] the impregnation method to load mesoporous silica with α-TOC was used. Briefly, they dissolved α-TOC in ethanol and added mesoporous silica to the solution. Then they dried at 35 °C for 16 h under reduced pressure to obtain a solid powder. Then, mesoporous silica loaded with α-TOC was added into PE-LD matrix by using an internal mixer. The pellets obtained were then fed into a co-rotating laboratory twin-screw extruder equipped with a sheet die, to produce the active antioxidant films. The results showed a slower antioxidant release of the tocopherol loaded into a silica substrate when compared to the samples containing free tocopherol. However, it also was demonstrated that the release of α-TOC depends on the size of the silica mesoporous.

Sun, Lu, Qiu, & Tang [84] reported a similar study using PE-LD films with α-TOC impregnated into silica mesoporous. They reported similar effects of α-TOC impregnated onto silica mesoporous on the release behavior of the antioxidant. Melendez-Rodriguez et al. [85] developed mesoporous silica nanoparticles loaded with eugenol by vapor absorption process. This process consisted in mixing 100 mg of eugenol with 100 mg of the mesoporous silica in a tightly closed vial. The mixture was incubated in an oven at 40 °C for 24 h while being continuously shaken. Thereafter, the loaded nanoparticles were incorporated into poly(3-hydroxybutyrate-co-3-hydroxyvalerate) (PHBV) by electrospinning, and electrospun composite fibers were produced. These fibers were subjected to annealing in a 4122-model press at 155 °C for 5 s, without pressure, and PHBV active films were produced. The antimicrobial performance against foodborne bacteria was evaluated, whereas it showed to inhibit bacterial growth successfully against *S. aureus* and *E. coli*.

Alkan, Sehit, Tas, Unal, & Cebeci [86] report the study of halloysite nanotubes (HNTs) loaded with carvacrol producing an aqueous solution and applying ultrasonication process. Then, carvacrol loaded halloysite was mixed with chitosan solution (positively charged) and poly (sodium-4-styrene sulfonate) (PSS) solution (negatively charged), and applied on PE film (not specified the type of PE) surface treated with plasma, using the spray LbL deposition technique. Then, antimicrobial activity in chicken meat samples packaged with the coated PE films was evaluated (type of packaging not specified). The results demonstrated that the microbiological quality of the samples packaged with PE films coated with HNTs loaded with carvacrol were significantly improved, when compared to samples packaged with neat PE films. This improvement in the microbiological quality means an increase in shelf-life for the food product.

Another option that has been used to incorporate active compounds are cyclodextrins (CDs). They are cyclic oligosaccharides capable of forming inclusion complexes with many organic compounds, including essential oils and volatiles, or their components. They can lead to changes in the solubility and reactivity of the added molecules. For example, Oliveira et al. [87] produced β-CS loaded with sorbic acid (SA) by molecular inclusion, which were added in the formulation of starch and poly (butylene adipate co-terephthalate) (PBAT). Then, the active films were produced using an extrusion process. They reported that CDs protected SA during film preparation, reducing its sublimation at high temperatures, and provided a controlled release of SA. However, the color of the film presented changes, and the concentration of SA was not efficient in controlling microorganisms.

Another strategy used is the incorporation, in packaging materials, of active compounds into fibers that are electrospun. The fibers are essentially produced by electrospinning with diameters that can range from nanometers to microns. To produce these fibers, a wide range of synthetic polymers, biopolymers, and biodegradable polymers are used. For example, Quiles-Carrillo et al., 2019, [38] studied PLA films coated with PLA electrospun structures loaded with gallic acid, used as an antioxidant agent. The PLA films were produced by cast film extrusion and the fibers loaded with gallic acid were deposited on surface of PLA film by the electrospinning process. They produced a PLA film coated with fibers (referred to as bilayer film) and also another film with fibers. Then they sandwiched both films with another PLA film on the other side (called multilayer). The antioxidant activity of gallic acid from PLA films was evaluated by the 2,2-diphenyl-1-picrylhydrazyl radical (DPPH) methodology. The results showed that during the first days, the bilayer PLA films showed more antioxidant activity than the multilayer films. However, the PLA films containing the electrospun multilayer achieved a stronger DPPH inhibition in a longer time.

Another example was presented by Figueroa-Lopez et al. [88] that produced an electrospun antimicrobial layer made of PHBV that was deposited on a previously produced PHA-based film. They combined OEO and zinc oxide nanoparticles (ZnO NPs) as antimicrobial agents in concentration of 2.5 and 2.25 wt.%, respectively, and produced the PHBV nanofibers. Then, they produced different active multilayer films, and the antimicrobial and antioxidant activity were evaluated. The multilayer films showed a significant inhibition of the antimicrobial activity (R) (R ≥ 1 and <3) against *S. aureus* and *E. coli* after 15 days. Concerning the antioxidant activity after 15 days, the multilayer films showed a reduction of DPPH inhibition due to the reduced release of the active compounds.

Chiralt, Tampau, & Gonz [89] developed electrospun fiber mats of PCL loaded with carvacrol. Afterwards, these fibers were deposited in between starch films in order to obtain multilayer films with a starch film/PCL loaded/starch film structure. The starch films were produced by melt blending of starch-glycerol-water at 160 °C and 8 rpm, for 30 min., in a two-roll mill. The multilayer structure was obtained using a thermocompression process. The barrier properties and antimicrobial activity were analyzed. The results showed that the film has an antimicrobial effect against *Escherichia coli* and a better barrier to water vapor.

In 2020, Figueroa-Lopez et al. [90] loaded OEO into α- and γ-CDs and incorporated them during electrospinning into poly(3-hydroxybutyrate-co-3-hydroxyvalerate) (PHBV) fibers. Afterwards, the loaded PHBV electrospun mats were subjected to annealing, and transparent films were produced. The antioxidant and antimicrobial activity were evaluated. The authors reported that antioxidant and antimicrobial activity for γ-CD inclusion complex was higher than for α-CD, because the OEO has higher solubility in the γ-CD and has bigger pore size.

The production of small capsules or nanoparticles for the incorporation of active agents is also possible by using different methodologies and materials. For example, Glaser et al. [91] used chitosan to produce nanocapsules loaded with resveratrol, by the ionic gelation technique. This technique allows the production of nano- and microparticles by ionic interactions between the two materials of different charges. Then, the solution with nanoparticles loaded with the active agent was applied to PE (not specified the type of PE) and PP surface (untreated and oxygen plasma treated), in two layers, by the LbL deposition technique. The effect of plasma treatment on films’ properties, and on antimicrobial and antioxidant activity, was evaluated. They observed that the O_2_ plasma treatment on the surface of PP and PE films before the coating improved the interaction between the two films and the coating with nanoparticles of chitosan. The results of active films functionalized with plasma treatment showed better properties and superior antimicrobial and anti-oxidative activity.

In 2011, Guarda, Rubilar, Miltz, & Jose [92] studied the antimicrobial activity of a BO-PP film coated with microcapsules produced by an emulsion of oil in water (O/W) containing thymol and carvacrol. For the production of microcapsules an aqueous solution of Arabic gum and a mixture of four different concentrations of the active agents with soybean oil (used as oil phase) was used. Then, the emulsion was homogenized at ambient temperature with high shear equipment. Before applying the coating on the BO-PP, the film surface was treated by corona, but the authors do not refer to the coating process used. The results demonstrated that antimicrobial activity was not altered by the microencapsulation, but the release rate of the active agents was lower and controlled when compared to the case where the active agents were incorporated directly into the matrix.

**Table 5:** Applications of carriers of the active agents added into flexible films.

Active Agent	Materials of Carrier	Material/Matrix	Packaging Function	Tested Product/Packaging Type	Loading of AA	Active Agent Amount	Main Effects Compared Control Film	References
Green tea	Crystallinemicroporous aluminosilicates	PE film	Antioxidant activity	Fresh minced meat/direct contact	6.4–12.8 mg/g of carrier material	20 and 40 wt.%	The results showed that active packaging developed extended the shelf life of fresh minced meat for 3 days when compared to a control sample.	[82]
α-TOC	Mesoporous silica	PE-LD film	Oxygen scavengers	-	α-TOC/silica weight ratio was 0.42—0.73	3 wt.%	The results demonstrated a slower release of α-TOC into a silica substrate (decrease about 60%) when compared to films samples with free tocopherol.	[83]
α-TOC	Mesoporous silica	PE-LD film	Antioxidant activity	-	Not specified	1 wt.%	The results exhibited radical scavenging activity of the active film, which increased from 28.45% to 46.50% during 24 h of DPPH test.	[84]
Eugenol	Mesoporous silica (MCM–41)	PHBV films	Antimicrobial activity	-	500 mg/g of MCM—41	2.5, 5, 7.5 and 10 wt.%	The electrospun PHBV films incorporated mesoporous silica nanoparticles with eugenol showed antimicrobial activity after 15 days.	[85]
Carvacrol	Halloysite nanotubes	PE film	Antimicrobial activity	Chicken meat/ns	-	15 wt.%	The results showed that the samples packaged with films developed with HNTs loaded with carvacrol decreased 85% in the viability of cells, demonstrating a strong bactericidal effect against *A. hydrophila.*	[86]
Sorbic acid (SA)	beta-cyclodextrin (β-CD)	PBAT film	Antimicrobial activity	-	100 mg SA/1 g β-CD	1 wt.%	The results showed that active films developed were not efficient in the control microorganisms, due to the low concentration (1% *w*/*w*) of active agent used in the film formulation.	[87]
Gallic acid	PLA fibers	PLA film	Antioxidant activity	-	40% based on the PLA weight	Not specified	The results showed that the PLA films containing the electrospun GA-loaded interlayer have a sustained release of the active agent for 10 weeks.	[38]
Oregano essential oil (OEO) and ZnO NPs	PHBV fibers	PHA film	Antimicrobial activity	-	Not specified	2.5 wt.% of OEO and 2.25 wt.% of ZnO NPs	The multilayer films developed showed a high antimicrobial and antioxidant activities in both open and closed systems for up to 15 days.	[88]
Carvacrol	PCL fibers	Starch film	Antimicrobial activity	-	12 g/100 g fibers	15 wt.%	The active film developed showed the antimicrobial effect against *E. coli*, but was not effective at controlling the growth of *Listeria innocua.*	[89]
Oregano essential oil (OEO)	alfa-and gamma-cyclodextrin (α- and ɣ-CD)	PHBV	Antioxidant and antimicrobial activity	-	Weight ratios of α-CD:OEO and γ-CD:OEO were 80:20 wt/wt and 85:15 wt/wt, respectively	10, 15, 20, 25, and 30 wt.%	The activity of films was evaluated during storage and it was observed that they are stable up to 15 days, which was explained by the protection offered by the developed system.	[90]
Resveratrol	Chitosan	PE and PP film	Antimicrobial and antioxidant activity	-	Not specified	2 wt.%	The active films showed over 90% reduction of *S. aureus* and over 77% reduction of *E. coli* as compared to untreated samples and increase antioxidant activity for over a factor of 10.	[91]
Carvacrol and thymol	Oil-in-water emulsion	BO-PP film	Antimicrobial activity	-	Not specified	1, 2, 5 and 10 wt.%	The results demonstrated that thymol and carvacrol microencapsulated and added on surface film were able to act for fresh food preservation against microorganisms, such as *E. coli* O157:H7, *S. aureus*, *L. innocua*, *Saccharomyces cerevisiae,* and *Aspergillus niger*.	[92]

**Legend: ns**—not specified if the active agent reacts by direct contact or headspace.

## 5. Conclusions and Future Trends

Active food packaging has been seen as a way to extend shelf-life and to maintain the sensory properties, quality, and safety of packaged food. In some cases, this type of packaging can also help to decrease the addition of preservative agents into foods. Among the active agents used to add this capability to the packaging, metallic particles already showed their capacity, while natural compounds still face several technological changes (e.g., sensitivity to temperature). Among the industrial production processes, incorporating active agents by extrusion is a challenge since the homogenous distribution of the active components in the matrix and the high temperatures used can limit the active agents’ performance. To overcome some of these issues, the scientific community and the industry have developed other strategies such as incorporating the active agents by coating technologies, which in most cases are commonly used by the industry. Nevertheless, some new methodologies, such as LbL technique and ultrasonic atomization, are expected to rise in the coming years as a solution for the development of active packaging. There are also more innovative strategies such as the supercritical fluid carbon dioxide or the use of carrier systems. For the last case, it is expected that micro and nanoencapsulation could bring some advantages. In fact, loading of active compounds in some of those systems can help to stabilize the compounds during the extrusion process, or the controlled release of the compounds when they are in contact with food.

It is also important to check the current legislation and the cost of all these new and innovative materials and technologies to assess if they can be easily implemented. For example, some of the solutions presented in this review are still at the research and development level, and their commercialization requires, in some cases, regulatory approval.

## Figures and Tables

**Table 1:** Overview of active packaging technologies, active agents used, their mechanisms and potential benefits in food applications.

**Active Scavenger System (or Absorber)**	**Classification**	**Materials**	**Mechanism**	**Potential Benefits**	**References**
Oxygen scavenger	Metallic and metallic oxides	Iron, ferrous oxide, cobalt, zinc, copper, magnesium, aluminum, titanium	Oxidation of metals with the supply of moisture and action of an optional catalyst.	Prevention of discoloration; prevention of mold growth; retention of vitamin C content, prevention of browning; prevention of rancidity.	[22,23,24,25,26]
	Inorganic	Sulfite, thiosulfate, dithionite, hydrogen sulfite, titanium dioxide	Oxidation of inorganic substrate by UV light.		
	Organic	Ascorbic acid, tocopherol, gallic acid, hydroquinone, catechol, rongalit, sorbose, lignin, pyrogallol, glucose oxidase, laccase	Oxidation of organic substrate with metallic catalyst or alkaline substance.		
	Polymer-based	Polymer metallic complex	Oxidation of polymer components with metallic catalyst (mostly cobalt).		
Moisture absorber	Inorganic	Silica gel (SiO_2_), potassium chloride (KCl), calcium chloride (CaCl_2_), sodium chloride (NaCl), calcium sulfate (CaSO_4_)	The common process is adsorption and absorption.	To control the moisture content in headspace of packaging and absorber of liquids.	[27,28,29,30,31]
	Organic	Sorbitol, fructose, cellulose and their derivatives (e.g., carboxymethylcellulose (CMC))			
	Polymer-based	Polyvinyl alcohol (PVOH) and sodium polyacrylate			
	Other synthesized	Synthesized attapulgite with acrylamide			
Ethylene scavenger	Minerals	Clays modified (e.g., MMT, organoclays, halloysite nanotubes (HNTs)) and zeolites, titanium dioxide (TiO_2_)	Adsorption process and cation exchange.	Reduction in ripening and senescence of fruits and vegetables.	[32,33,34,35]
	Metallic and metallic oxides	Silver (Ag) and zinc oxide (ZnO)	Activated by either UV light, visible light or both.		
Active Releaser system (or emitter)	Classification	Materials	Mechanism	Potential benefits	References
Antioxidants	Organic	Tocopherol, carvacrol, quercetin, catechin, thymol, gallic acid, ascorbic acid, rosemary, green tea, oregano, cinnamon, sage leaf and bay leaf extracts, eugenol, olive leaf, mango leaf	Free radicals and peroxides react to retard or block the actual oxidation reactions.	Prevention of fat oxidation and food deterioration maintenance of nutritional quality, texture and functionality.	[36,37,38]
	Metallic and metallic oxides, and inorganic	Silver (Ag), copper (Cu), titanium dioxide (TiO_2_) and zinc oxide (ZnO)	Catalytic function that reduces the rate oxidation.		
Antimicrobials	Organic	Allyl- isothiocyanate, cinnamaldehyde, carvacrol, thymol, eugenol, oregano, basil leaf, extract of allium, lauric arginate ester, sodium lactate, sorbic acid, citric acid	Metabolic and reproductive processes of microorganisms are blocked or inhibited. Cell wall conformation modification.	Inhibition of spoilage and retardation of pathogenic microorganism’s growth.	[8,39,40]
	Polymers	Chitosan and ε-Polylysine			
	Enzymes, bacteriocins and antibiotics	Lysozyme, lactoferrin, nisin, lactocins, pediocin, enterocins			
	Metallic and metallic oxides, and inorganic	Silver (Ag), copper (Cu), titanium dioxide (TiO_2_) and zinc oxide (ZnO)			

## Data Availability

Not applicable.

## Abbreviations

ALD	Atomic layer deposition
Al_2_O_3_	Aluminum oxide
Ag	Silver
Ag NPs	Silver nanoparticles
AgZeo	Silver substituted zeolite
Al	Aluminum
BHT	Butylated hydroxytoluene
BHA	Butylated hydroxyanisole
BioPE	Biobased linear low-density polyethylene
BO-PLA	Biaxially oriented polylactic acid
BO-PP	Biaxially oriented polypropylene
CA	Citric acid
CaO	Calcium oxide
Chit	Chitosan
CuO	Copper oxide
DPPH	2,2-diphenyl-1-picrylhydrazyl radical
EC	European Commission
EU	European Union
EVA	Ethylene vinyl acetate copolymer
EVOH	Ethylene vinyl alcohol
PE-HD	High-density polyethylene
HNTs	Halloysite nanotubes
KMnO_4_	Potassium permanganate
LA	Lactic acid
LbL	Layer-by-layer
PE-LD	Low-density polyethylene
LevA	Levulinic acid
PE-LLD	Linear low-density polyethylene
MAP	Modified atmosphere packaging
NPs	Nanoparticles
Nis	Nisin
OEO	Oregano essential oil
OPP	Bioriented polypropylene
OS	Oxygen scavenger
PA	Polyamide
PAA	Poly(acrylic acid)
PBAT	Poly (butylene adipate-coterephthalate)
PCL	Poly-(ε-caprolactone)
PE	Polyethylene
PEI	Polyethyleneimine
PEO	Poly(ethylene oxide)
PE-g-MA	Polyethylene-grafted maleic anhydride
PP	Polypropylene
PET	Polyethylene terephthalate
PHAs	Polyhydroxyalkanoates
PHBV	Poly(3-hydroxybutyrate-co-3-hydroxyvalerate)
PLA	Poly(lactic acid)
PS	Potassium sorbate
PSS	Poly (sodium-4-styrene sulfonate)
PVA	Poly(vinyl alcohol)
PVdC	Poly(vinylidene chloride)
R	Value of the antimicrobial activity
RH	Relative humidity
RTE	Ready-to-eat meat
SC-CO_2_	Supercritical impregnation process by carbon dioxide
SFCs	Supercritical fluids
TBARS	Thiobarbituric acid reactive substance
TBHQ	Tert-butylhydroquinone
TPP	Sodium tripolyphosphate
TPS	Thermoplastic starch
TiO_2_	Titanium dioxide
UV	Ultraviolet
ZnO	Zinc oxide
α-TOC	Alfa-tocopherol
